# Safety assessment on comedogenicity of dermatological products containing d-alpha tocopheryl acetate in Asian subjects: A double-blind randomized controlled trial

**DOI:** 10.1016/j.conctc.2021.100834

**Published:** 2021-08-19

**Authors:** Neti Waranuch, Sutthinee Wisutthathum, Supaporn Tuanthai, Pichet Kittikun, Francois Grandmottet, Francine Tay, Jarupa Viyoch

**Affiliations:** a Department of Pharmaceutical Technology, Faculty of Pharmaceutical Sciences, Naresuan University, Phitsanulok, 65000, Thailand; b Cosmetics and Natural Products Research Center, Faculty of Pharmaceutical Sciences Naresuan University, Phitsanulok, 65000, Thailand; c Center of Excellence for Innovation in Chemistry, Naresuan University, Phitsanulok, 65000, Thailand; d Department of Biochemistry, Faculty of Medical Science, Naresuan University, Thailand; e Blackmores Institute, 20 Jubilee Avenue, Warriewood, NSW, 2102, Australia

**Keywords:** Comedogenicity, Cosmetics, D-Alpha tocopheryl Acetate

## Abstract

A double-blind randomized controlled trial was used to assess the comedogenic potential of the dermatological products containing d-Alpha tocopheryl acetate. A total of 15 healthy males (20–45 years old) with prominent follicular orifices and the ability to form comedones on the upper aspect of the back were enrolled. Each participant was given pads containing 4 test products. The positive control arm received a pad containing octyl palmitate which is a reported comedogenic material. The negative control arm received a pad without any test material. Participants were randomized to apply either the positive, negative or the active test cream to the application area for 4 weeks. Comedones were identified using epidermal biopsy under a stereomicroscope. The average number of microcomedone before exposure (baseline) with octyl palmitate was 6.1 ± 0.6 (mean ± SEM), and changed to 27.3 ± 4.7 which was more than 50% increase in comedone formation in every subject with the average change from base line was 365.4 ± 87.6%. In the negative control arm the average number of microcomedone at baseline was 6.4 ± 1.1 and at 4 week-application was 3.4 ± 0.6 (−43.0 ± 9.5% increased). All tested products produced less than a 50% increase in the number of microcomedones. Analyzed data from 12 subjects indicated non-comedogenic potential of the tested products containing-alpha tocopheryl acetate and other ingredients including lanolin, kernel oil and avocado oil and sunflower oil, etc. The octyl palmitate produced more than 50% increase in comedone formation in every analyzed subject.

## 1 Introduction

Dermatological products are referred to topical prescriptions, over-the counter or cosmetic products applying to the human body for treatment, cleansing, beautifying, promoting attractiveness or altering the appearance without affecting body structure or functions. However, they can be the cause of skin disorders of varying severity, namely irritation, folliculitis, contact dermatitis, photosenstization and comedones. The term comedogenicity refers to the potential of various agents to promote the abnormal keratinization (hyperkeratinization) and desquamation of follicular epithelium [1]. These abnormalities lead to a partial (open comedone or blackhead) or complete obstruction of the pilosebaceous (closed comedone or whitehead) and accumulation of sebum.

The comedogenic potential of dermatological products has been documented since 1972 by Kligman AM and Mills OH [1]. The comedogenic activity of the dermatological ingredients, for example, apricot kernel seed oil, cocoa butter, corn oil, isopropyl myristate, mineral oil, acetylated lanolin, octyl palmitate, sunflower oil, sodium lauryl sulfate, tocopherol etc. has been listed [[1], [2], [3]]. These results were conducted from testing of 100% concentration of the tested ingredients in animal models, namely rabbit ear assay. However, the comedogenic potential of such ingredients cannot be taken to be the same as finished products, as the mixtures of ingredients and application to human skin will alter the final comedogenicity of each product. For this reason, it is desirable for the comedogenicity of finished products to be documented through additional clinical observations.

In the present study, the comedogenicity of the marketed dermatological products were assessed by using human model modified from the previous study [3,4]. All tested products contained **d-Alpha tocopheryl acetate** in an amount 5 IU/100 g of product, as a main active ingredient. The formula also contained *Brassica campestri*s (Aleurites fordi) oil, Arginine PCA (and) *Phaeodactylum tricormutum* extract, *Prunus armeniaca* (apricot) kernel oil, *Persea gratissima* (avocado) oil, Retinyl palmitate (and) *Helianthus annuus* (sunflower) seed oil, Lanolin, *Crocus chrysanthus* bulb extract (and) Acacia senegal gum and *Callophyllum inophyllum* seed oil, as an alternative active. Additionally, other ingredients including glycerin, lanolin, cetyl alcohol, myristyl myristate, polysorbate 60, sorbitan stearate, phenoxyethanol, hydroxyacetophenone, glyceryl stearate SE, linoleic acid & linolenic acid, triethanolamine, carbomer, tocopherol were used as basic excipients.

Focusing on an individual ingredient, D-alpha tocopheryl acetate which acts as the major active ingredient is known to be non-comedogenic [5], however there may be a possibility of forming other substances during production which may lead to comedogenicity within the finished product. Therefore, a clinical study to assess the comedogenicity of the tested product, including ingredients with non or weak comedogenic potential is prudent to ensure the consumer satisfaction.

## 2 Material and methods

### 2.1 The tested products

The marketed Natural Vitamin E Cream (emulsion form) for face and body, which were produced by Blackmores Ltd, Australia, including 1) Blackmores Natural Vitamin E Cream - **Skin Barrier** (actives: d-Alpha tocopheryl acetate, Brassica campestris (Aleurites fordi) oil and water (and) Arginine PCA (and) *Phaeodactylum tricormutum* extract; excipients: purified water, glycerin, lanolin, cetyl alcohol, myristyl myristate, polysorbate 60, grape seed oil, sorbitan stearate, avocado oil, (apricot) kernel oil, phenoxyethanol, hydroxyacetophenone, glyceryl stearate SE, retinyl palmitate (and) sunflower seed oil, linoleic acid & linolenic acid, tristhanolamine, carbomer, tocopherol) 2) Blackmores Natural Vitamin E Cream **PABA free** (actives: d-Alpha tocopheryl acetate, *Prunus armeniaca* (apricot) kernel oil, *Persea gratissima* (avocado) oil, Retinyl palmitate (and) *Helianthus annuus* (sunflower) seed oil; excipients: purified water, glycerin, lanolin, cetyl alcohol, myristyl myristate, polysorbate 60, sorbitan stearate, phenoxyethanol, hydroxyacetophenone, glyceryl stearate SE, linoleic acid & linolenic acid, triethanolamine, carbomer, tocopherol) 3) Blackmores Natural Vitamin E Cream **+ Lanolin** (actives: d-Alpha tocopheryl acetate, Lanolin; excipients: purified water, glycerin, cetyl alcohol, myristyl myristate, polysorbate 60, sorbitan stearate, avocado oil, (apricot) kernel oil, phenoxyethanol, hydroxyacetophenone, glyceryl stearate SE, retinyl palmitate (and) sunflower seed oil, linoleic acid & linolenic acid, triethanolamine, carbomer, tocopherol) and 4) Blackmores Natural Vitamin E Cream - **Firm & Smooth** (actives: d-Alpha tocopheryl acetate, *Crocus chrysanthus* bulb extract (and) Acacia senegal gum (and) water and *Callophyllum inophyllum* seed oil; excipients: purified water, glycerin, lanolin, cetyl alcohol, myristyl myristate, avocado oil, (apricot) kernel oil, polysorbate 60, sorbitan stearate, phenoxyethanol, hydroxyacetophenone, glyceryl stearate SE, retinyl palmitate (and) sunflower seed oil, linoleic acid & linolenic acid, triethanolamine, carbomer, tocopherol) were the tested products. Octyl palmitate (Cosmetics grade, Namsiang Co., Ltd.) was used as a positive control.

### 2.2 Study design

This study was conducted in accordance with the Declaration of Helsinki Principles. The study protocol was approved by the institutional review board (IRB No. 0030/62, COA No. 101/2019, Date of Approval: May 29, 2019) of Naresuan University, Phitsanulok, Thailand. The design of the study was a double-blind randomized controlled trial and was conducted from July 19 to August 28, 2019 at Cosmetics and Natural Products Research Center (CosNat), Naresuan University.

A total of 15 healthy males (20–45 years old) with prominent follicular orifices or visible comedones on the upper aspect of the back were the targeted number of subjected to enroll. Each enrolled subject was given pads containing the tested products to apply to the upper aspect of the back. The positive control was the pad containing octyl palmitate which has been reported as a comedogenic material [3,4]. The negative control was a pad without any material. Each participant was randomized to apply positive, negative or the active tested products by using a computer-generated random number. The study period was 4 weeks. The number of comedones were identified from epidermal biopsy under a stereomicroscope.

### 2.3 Study population

Healthy Thai male subjects aged between 20–45 years were eligible for the study. Subjects displaying prominent follicular orifices or visible comedones (open or closed comedone) on the upper aspect of the back were firstly recruited. Subjects were excluded if they had a scar in the study designed area, had a known allergy, photo-sensitization or hypersensitivity to cosmetics or dermopharmaceutical products, using systemic hormones, steroids, retinoids, immunosuppressive drugs, antibiotics, or using any of the topical acne treatment product on the back.

### 2.4 Study procedure

The volunteers were initially self-screened through advertising criteria. Eligible volunteers were informed of the project detail by investigators and asked to sign an informed consent before interviewing according to inclusion and exclusion criteria. Only volunteers who met the criteria enrolled to be the subjects of this study. Subjects continued to be recruited into the study until at least 15 subjects enrolled.

The assigned pad saturated with 0.2–0.5 mL of the tested products was applied to the tested site (back) 3 times/week. The total number of tested sites was 6 (4 for the tested products, 1 for the positive control and 1 for the negative control). The tested sites, each 4 × 4 cm were covered with a piece of non-absorbing pad (cotton cloth) that was closely adhered to the skin by occlusive hypoallergenic tape. The pads were removed after 48 h exposure, if they were placed on Monday and Wednesday, and 72 h exposure if they were placed on Friday. Upon removal, the tested sites were cleaned and assessed for any signs of undesirable effects prior to re-covering. This procedure was repeated weekly for 4 weeks. The epidermal biopsy specimen was performed on the tested sites both before patching (Day 1) and post-patching (15 min after last patch removal) by using 2 hypoallergenic strip pads to cover one test site (2 pieces/site, each piece 2 × 4 cm). Each strip pad was microscopically examined to determine the number of microcomedones.

### 2.5 Subject's study visit compliance

All subjects were detailed and the appointment schedule explained to them. All appointments were scheduled for arrival at the setting (CosNat) before 9.00 a.m. two or three days before an appointment date, all subjects were contacted to confirm an appointment time and place. If subjects were unable to meet the appointment, a new visit within 1–2 days before or after the previous appointment was rescheduled.

### 2.6 Determination of skin tolerance

The tested products used in this study were composed of well recognized dermatological ingredients. However, a long duration of test application may cause undesirable side effects such as itching, stinging, redness, rash, edema, scaling, eczema, burn and, hypersensitive from light.

Visual grading scales by dermatologist were used to evaluate undesirable effects of the tested products. The grading scale ranged from 0 to 3. Zero was defined as non-reaction, 1 was defined as mild, 2 was defined as moderate, and 3 was defined as severe. Types of skin reactions evaluated include itching, stinging, redness, rash, edema, scaling, eczema, burn and, hypersensitive from light.

The negative control should provide less than a 50% increase in microcomedone number. Positive control of known comedogenic materials should produce a 50%–100% increase in microcomedone number, and the tested product that had produced less than a 50% increase in microcomedone number was considered non-comedogenic [3].

If any subject had shown a greater than 50% or less than 50% increase in microcomedone number after application of negative or positive control, respectively, the data from such subject were not included.

### 2.7 Statistical analysis

The data are expressed as descriptive statistic in terms of mean ± SEM of the number of comedones, frequency, and the percent change in microcomedone formation from baseline.

## 3 Results

### 3.1 Flow of subjects through the study

**Fig. 1:**
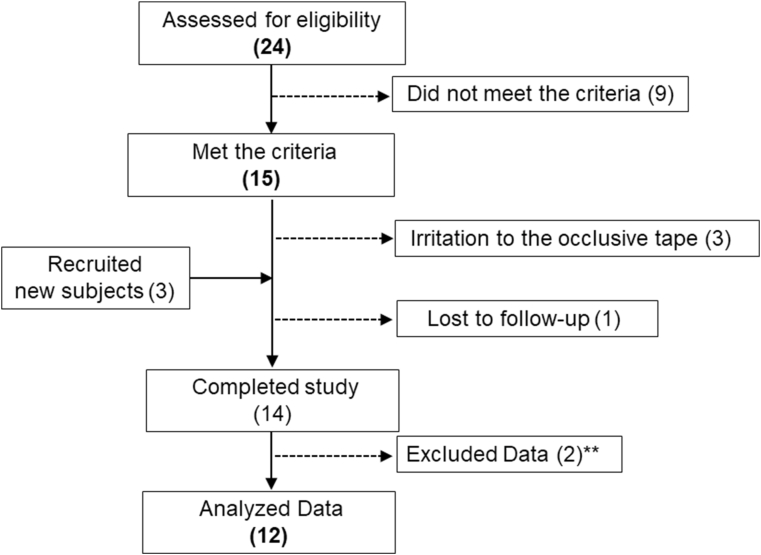
Flow of subjects through the study.

Twenty-four Thai volunteers signed informed-consent forms. Nine volunteers did not meet the criteria. As a result, a total of 15 subjects were enrolled. During the study for 2 and 3 weeks, 2 and 1 subjects respectively developed an irritation sign on the back areas that were directly in contact with the occlusive hypoallergenic tape. As a result, 3 new subjects were proceeded for recruitment. Fourteen subjects completed the study. One subject discontinued before study completion with his own personal reason that not involved with this study. Flow of subjects through the study is shown in Fig. 1.

### 3.2 Demographic data

Table 1 shows the demographic of fourteen subjects who completed the study. The age range of subjects was 22–43 years (35 years in average). Most subjects were working as the university employees (71.44%).

**Table 1:** Demographic data of fourteen subjects who completed the study for 4 weeks.

Male Subjects
Age range: 23–43 years
Age in Average: 35 ± 6 years
Occupation
University Employee: 10 (71.44%)
University Student: 1 (7.14%)
Research Assistance: 1 (7.14%)
Employee: 1 (7.14%)
Agriculturist: 1 (7.14%)

### 3.3 Comedogenicity

From the 14 subjects who completed the study for 4 weeks, one subject (7.1%) showed a greater 50% (66.7% as compared to baseline) and another one subject (7.1%) showed less than 50% (37.5% as compared to baseline) increase in microcomedone number after application of negative or positive control, respectively. Therefore, their data were not included for analysis.

**Fig. 2:**
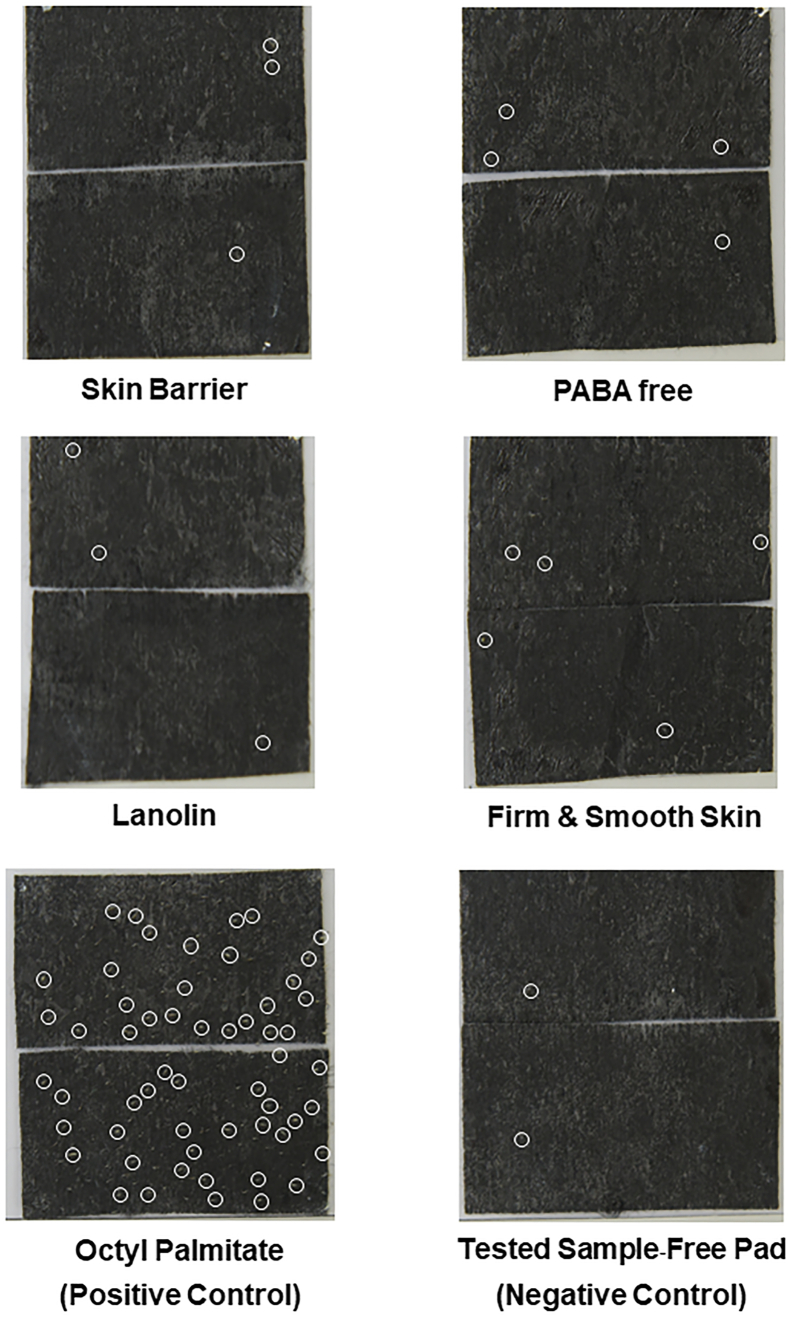
Microcomedones adhered to the strip pad after 4 weeks of exposure.

Table 2 presents analyzed data of twelve subjects obtained from each of the tested products. Data are expressed as the mean of number of microcomedones at both before and after product application. Table 3 presents the % subjects, mean of %change and SEM that responding to the test samples in each group i.e., increase, decrease and not-change. For positive control (octyl palmitate), the average number of microcomedone at before testing (baseline) was 6.1 ± 0.6 (mean ± SEM). After 4 weeks of application, the mean ± SEM changed to 27.3 ± 4.7. The positive control arm produced more than 50% increase in comedone formation in every subject, and the average change from baseline was 365.4 ± 87.6%. For the negative control arm, 91.67% of subjects (11 of 12 subjects) decrease or not change in the number of microcomedones. The number of microcomedone at baseline averaged 6.4 ± 1.1 and at the 4 week-application was 3.4 ± 0.6 (−43.0 ± 9.5% increase). All tested products produced, 66.7–75% of subjects (8–9 of 12 subjects) decrease or not change in the number of microcomedones, and less than a 50% increase in the number of microcomedones in 25–33.3% of subjects (3–4 of 12 subjects). Overall subjects the percentage of microcomedones changing less than a 50% (%change in average: 13.8 ± 11.1% for **Skin Barrier**; −14.0 ± 11.8% for **PABA free**; −24.3 ± 12.0% for **+ Lanolin**; −25.6 ± 9.9% for **Firm & Smooth**, respectively), therefore, all tested products are considered non-comedogenic, according to the assessment criteria mentioned above. Fig. 2 shows number of microcomedones of each subject, Table 2, Table 3 show percentage of microcomedones adhered with the strip pads before and after the application sites were exposed to the tested products for 4 weeks. Moreover, the number of microcomedones tended to decrease in every tested group.

**Table 2:** The number of microcomedones in each subject before (B) and after (A) 4 weeks application of the tested products.

SubjectCode	Tested Samples																	
	Skin Barrier			PABA free			+Lanolin			Firm & Smooth			Octyl Palmitate (Positive Control)			Tested Sample-Free Pad (Negative Control)		
	No. of Microcomedones			No. of Microcomedones			No. of Microcomedones			No. of Microcomedones			No. of Microcomedones			No. of Microcomedones		
	B	A	% Increase	B	A	% Increase	B	A	% Increase	B	A	% Increase	B	A	% Increase	B	A	% Increase
02	6	7	16.7	5	4	−20.0	5	4	−20.0	4	5	25.0	5	17	240.0	5	4	−20.0
03	7	7	0.0	6	6	0.0	7	8	14.3	4	5	25.0	8	42	425.0	6	6	0.0
04	9	7	−22.2	10	13	30.0	10	13	30.0	17	11	−35.3	10	51	410.0	18	8	−55.6
06	5	7	40.0	5	7	40.0	4	4	0.0	5	6	20.0	4	8	100.0	4	5	25.0
08	5	5	0.0	3	3	0.0	4	5	25.0	5	5	0.0	4	44	1000.0	5	4	−20.0
10	4	2	−50.0	4	2	−50.0	4	1	−75.0	4	2	−50.0	4	9	125.0	4	2	−50.0
11	4	2	−50.0	4	1	−75.0	5	3	−40.0	5	3	−40.0	5	10	100.0	4	2	−50.0
12	9	12	33.3	7	7	0.0	6	4	−33.3	9	7	−22.2	9	15	66.7	6	2	−66.7
19	5	4	−20.0	5	6	20.0	6	2	−66.7	6	3	−50.0	6	36	500.0	5	1	−80.0
24	9	11	22.2	6	3	−50.0	7	2	−71.4	8	3	−62.5	7	15	114.3	9	4	−55.6
25	4	1	−75.0	5	1	−80.0	4	1	−75.0	6	3	−50.0	5	46	820.0	5	2	−60.0
28	5	2	−60.0	6	7	16.7	5	6	20.0	6	2	−66.7	6	35	483.3	6	1	−83.3
**Mean**	**6.0**	**5.6**	**−13.8**	**5.5**	**5.0**	**−14.0**	**5.6**	**4.4**	**−24.3**	**6.6**	**4.6**	**−25.6**	**6.1**	**27.3**	**365.4**	**6.4**	**3.4**	**−43.0**
**SEM**	**0.6**	**1.0**	**11.1**	**0.5**	**1.0**	**11.8**	**0.5**	**1.0**	**12.0**	**1.0**	**0.7**	**9.9**	**0.6**	**4.7**	**87.6**	**1.1**	**0.6**	**9.5**

**Table 3:** The percentage change of microcomedones and number of subjects responding after 4 weeks application of the tested products.

Microcomedones Changing	Skin Barrier			PABA free			+Lanolin			Firm & Smooth			Octyl Palmitate (Positive Control)			Tested Sample-Free Pad (Negative Control)		
	n (%)	% Change	SEM	n (%)	%Change	SEM	n (%)	%Change	SEM	n (%)	%Change	SEM	n (%)	%Change	SEM	n (%)	%Change	SEM
Increase	4 (33.3)	28.1	5.3	4 (33.3)	26.7	5.3	4 (33.3)	22.3	3.4	3 (25)	23.3	1.7	12	365.4	87.6	1 (8.3)	25	0
Decrease	6 (50)	−46.2	8.8	5 (41.7)	−55.0	10.7	7 (58.3)	−54.5	8.6	8 (66.7)	−47.1	5.1	–	–	–	10 (83.3)	−54.1	6.7
Not-change	2 (16.7)	–	–	3 (25)	–	–	1 (8.3)	–	–	1 (8.3)	–	–	–	–	–	1 (8.3)	–	–
Overall	12 (100)	−13.8	11.1	12 (100)	−14.0	11.8	12 (100)	−24.3	12.0	12 (100)	−25.6	9.9	12 (100)	365.4	87.6	12 (100)	−43.0	9.5

### 3.4 Cutaneous tolerance

**Fig. 3:**
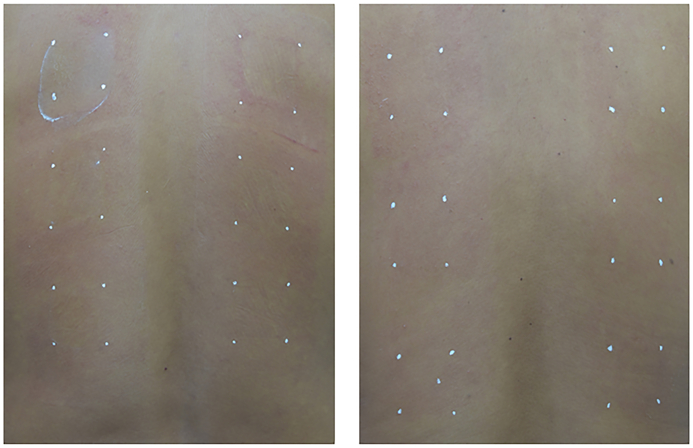
Skin redness appearance on the areas that directly in contact with the occlusive.

During the study for, three subjects were discontinued from the study as their back areas that directly in contact with the occlusive hypoallergenic tape developed irritation. Fig. 3 shows the examples of skin redness appearance of two subjects who their backs were exposed to the hypoallergenic tape for 2 weeks. Such redness disappeared within 1 or 2 days after ceasing exposure.

For the areas exposed to the tested products, all subjects reported no severe unwanted effects. After application of the tested products, including the negative control, minimal erythema and dryness were found in some subjects. This may be caused by the occlusive effect. From the obtained results, all tested products were well tolerated for cutaneous exposure, according to this designed study.

## 4 Discussion

According to the ban on animal testing for many products, nowadays the human model has been developed and accepted for comedogenic testing of finished products. Comedones counting can be performed by various skin imaging techniques and correlated to skin surface characteristics (microrelief) [6]. The principle of the comedogenic testing on the human model is to investigate the level or number of microcomedones after applying the designed amount of the tested product on the back of subjects with comedone prone skin.

In the present study, the conducted methodology was adopted from the previous studies [3,4], which tested on the upper aspect of the back. It was recommended that the test site should be related to the characteristics of the finished products due to the low correlation in comedogenic response of different areas [7]. It is interesting to note that small numbers of subjects were enrolled in the previous studies. Therefore, the sample size may not be a major factor of concern for testing the comedogenic potential but skin type with notable follicular orifices or comedone prone is the important criteria for subject inclusion.

In this study, 15 subjects with clear follicular orifice on their backs was the targeted number of subjects to enroll, and octyl palmitate was employed as a positive control. When this ingredient was applied under occlusion to the back of Asian subjects for 4 weeks, 14 subjects who completed the study showed an increase in the number of microcomedone. This indicates that octyl palmitate is an appropriate positive control for comedogenicity test under the condition used. The mechanism(s) of octyl palmitate to induce comedone has not been clarified yet. In fact, the comedone involves either hyperkeratinization of the follicular epithelium [8] or delayed desquamation of horny cells [9]. The mechanism in action differs in details among various ingredients.

Generally, acetylated lanolin and isopropyl myristate (IPM) has been mainly used as a positive control in animal [10] and human models [3]. However, it has been suggested that IPM may not be a good positive control for comedogenicity test in human studies, particularly in Asian subjects having lower comedogenicity sensitivity than Caucasians [4].

From our study, however, as compared to other subjects, one subject showed a small increase in the number of microcomedones (37.5%) at the site applied with octyl palmitate, and another one subject showed more than 50% increase (66.7%) at the site covered with the pad without any material (negative control). This depicts a subject variability. Therefore, the data from these subjects were excluded in analysis, and the data from 12 subjects in total were analyzed for an averaged number of microcomedone and percent increase.

Focusing on individual ingredient in the formulations, all ingredients used have been classified as being of non or weak comedogenic potential, according to study in animal or human model [[2], [3], [4]]. Moreover, they have provided various beneficial effects to the skin. For examples, it has been reported that antioxidants and vitamins, including Vitamin E have a potential in the prevention of acne inflammation through a reduction of peroxide formation [11]. Natural lanolin combined with avocado oil, apricot kernel oil and sunflower are also able to protect the skin surface from dehydration, thus possible reducing the pores clogged from an excessive sebum production [12,13]. Even though each single ingredient is unlikely to be comedogenic in human, the tested products may or may not produce comedone on the application site. It is possible that a formation of comedogenic substance(s) due to chemical interaction between the ingredients used occurs during the emulsification process. For this reason, a comedogenic assessment of the finished products should be investigated to ensure product safety and performance. In the present study, after a 4-week application of the tested products under occlusive condition, 66.7–75% of subjects (8–9 of 12 subjects) decrease or not change the number of microcomedone, and 25–33.3% of subjects (3–4 of 12 subjects) increase in number of microcomedones, which percentage increase was less than 50%. Overall subjects were analyzed an averaged number of microcomedones and percent increase, these data indicated that all tested products produced less than a 50% increase in the number of microcomedones. Therefore, the tested products are considered non-comedogenic, according to the assessment criteria.

It is important to emphasize that our study design under occlusive condition for 4 weeks is not indicative of the normal use of products under non-occlusion for several weeks or months. However, the results obtained from the exaggerated design may be applicable to the large Asian population.

## 5 Conclusion

There are many reports of causing comedones. This undesirable reaction is of great concern to physicians and users. Therefore, comedogenicity testing of finished products is prudent. A variety of methods have been used to evaluate comedones formation using both animal and human models. In the present study, the study in human with prominent follicular orifices or visible comedones on the upper aspect of the back was designed. The pad saturated with 0.2–0.5 mL of the tested products was delivered to the tested site (back) 3 times/week for 4 weeks. The tested products contained d-Alpha tocopheryl acetate and other ingredients including lanolin, kernel oil and avocado oil and sunflower oil, etc. The positive control was octyl palmitate, and the negative control was a pad without any test material. The number of microcomedones were observed from epidermal biopsy under a stereomicroscope. The analyzed data from 12 subjects indicated non-comedogenic potential of the finished products tested. The positive control produced more than 50% increase in comedone formation in every analyzed subject.

## Declaration of competing interest

The authors declare that they have no known competing financial interests or personal relationships that could have appeared to influence the work reported in this paper.
